# Genomic and systems evolution in *Vibrionaceae *species

**DOI:** 10.1186/1471-2164-10-S1-S11

**Published:** 2009-07-07

**Authors:** Jianying Gu, Jennifer Neary, Hong Cai, Audrey Moshfeghian, Stephen A Rodriguez, Timothy G Lilburn, Yufeng Wang

**Affiliations:** 1 Department of Biology, College of Staten Island, City University of New York, Staten Island, NY 10314, USA; 2 Department of Biology, University of Texas at San Antonio, San Antonio, TX 78249, USA; 3 Department of Bacteriology, American Type Culture Collection, Manassas, VA 20110, USA; 4 South Texas Center for Emerging Infectious Diseases, University of Texas at San Antonio, San Antonio, TX 78249, USA

## Abstract

**Background:**

The steadily increasing number of prokaryotic genomes has accelerated the study of genome evolution; in particular, the availability of sets of genomes from closely related bacteria has facilitated the exploration of the mechanisms underlying genome plasticity. The family *Vibrionaceae *is found in the *Gammaproteobacteria *and is abundant in aquatic environments. Taxa from the family *Vibrionaceae *are diversified in their life styles; some species are free living, others are symbiotic, and others are human pathogens. This diversity makes this family a useful set of model organisms for studying bacterial evolution. This evolution is driven by several forces, among them gene duplication and lateral gene transfer, which are believed to provide raw material for functional redundancy and novelty. The resultant gene copy increase in one genome is then detected as lineage-specific expansion (LSE).

**Results:**

Here we present the results of a detailed comparison of the genomes of eleven *Vibrionaceae *strains that have distinct life styles and distinct phenotypes. The core genome shared by all eleven strains is composed of 1,882 genes, which make up about 31%–50% of the genome repertoire. We further investigated the distribution and features of genes that have been specifically expanded in one unique lineage of the eleven strains. Abundant duplicate genes have been identified in the eleven *Vibrionaceae *strains, with 1–11% of the whole genomes composed lineage specific radiations. These LSEs occurred in two distinct patterns: the first type yields one or more copies of a single gene; we call this a single gene expansion. The second pattern has a high evolutionary impact, as the expansion involves two or more gene copies in a block, with the duplicated block located next to the original block (a contiguous block expansion) or at some distance from the original block (a discontiguous block expansion). We showed that LSEs involve genes that are tied to defense and pathogenesis mechanisms as well as in the fundamental life cycle of *Vibrionaceae *species.

**Conclusion:**

Our results provide evidence of genome plasticity and rapid evolution within the family *Vibrionaceae*. The comparisons point to sources of genomic variation and candidates for lineage-specific adaptations of each *Vibrionaceae *pathogen or nonpathogen strain. Such lineage specific expansions could reveal components in bacterial systems that, by their enhanced genetic variability, can be tied to responses to environmental challenges, interesting phenotypes, or adaptive pathogenic responses to host challenges.

## Background

The diversity and ubiquity of members of the domain Bacteria is convincing evidence of their ability to develop successful adaptive strategies during evolution. Evolutionarily closely related species or strains that have undergone lifestyle changes (such as from free-living to intracellular obligate pathogen, or from free-living to symbiotic) are excellent targets for studying genome plasticity and adaptive changes in bacterial systems.

Three principle mechanisms are considered important in bacterial adaptation: acquisition of new genetic material by lateral gene transfer or gene duplication, positive selection resulting in fixation of advantageous mutations in genes, and changes in gene expression regulation. Among the various evolutionary forces driving genome plasticity, gene duplication and lateral gene transfer are believed to provide raw material for functional redundancy and novelty in the development of biological systems in bacteria [1]. Gene duplication can arise from large scale (genome or chromosome block) duplications, small scale single gene duplications, nonhomologous recombination, or through the action of mobile genetic elements. When a gene duplication event occurs, the duplicate genes supply redundant functions. Deleterious mutations in one copy of a gene may be harmless because the redundant gene copy can provide a back-up function. Novelty can arise when one gene receives most of the selective pressure and shields the other copy, allowing it to evolve under relaxed selective constraints. The consequent elevated evolutionary rates are postulated to give rise to novel functions. Bacteria can also acquire new genes from other organisms via lateral gene transfer using the mechanisms of conjugation, bacterial phage infection, and acquisition of plasmids. The subsequent expansion of these new genes can increase the number of gene copies. The emergence of multiple gene copies by duplication or lateral gene transfer in a specific lineage is known as a lineage specific expansion (LSE) event [2].

The recent release of the complete genomic sequences for several *Vibrionaceae *strains provides an ideal model system for comparative studies of evolutionary mechanisms linked to different life styles and varying levels of pathogenicity [3-8]. The family *Vibrionaceae *is found in the *Gammaproteobacteria *and is abundant in aquatic environments. Members of this family are pathogenic for shellfish, finfish, other marine animals and humans. *Vibrio cholerae *is the etiological agent of cholera, which has claimed millions of lives over the centuries. This free-living pathogen can be found in seawater around the world. In the Ganges delta, it causes annual epidemics; the wave of infections is correlated with seasonal changes in rainfall and sunlight [9]. Complete genomic sequences are available for the serogroup O1 biogroup El Tor strain N16961, a toxigenic strain capable of causing epidemic cholera, and serogroup O1 biogroup Classical strain O395, which has been extensively used for molecular analysis. Two other members of this family, *V. parahaemolyticus *and *V. vulnificus*, do not cause epidemics; they are the causes of seafood-associated food poisoning. Genome sequences are available for *V. parahaemolyticus *strain RIMD 2210633, and two strains of *V. vulnificus*: YJ016 and CMCP6. Another two members of this family, *V. harveyi *and *V. splendidus*, are common in marine environments and are not human pathogens. *V. harveyi *is an opportunistic pathogen or a primary pathogen of many commercially cultured invertebrate species [10]. *V. splendidus *can cause disease and death in many marine species including commercially important fishes, oysters, mussels, and scallops. This bacterium was linked to significant mortalities in oysters (*Crassostrea gigas*) during the summer of 2001 [11]. Genome sequences are available for *V. harveyi *strain ATCC BAA-1116 and *V. splendidus *strain LGP32. A sixth member of this family, *Aliivibrio (Vibrio) fischeri*, is a non-pathogenic, bioluminescent symbiont living within the light emitting organs of the squid *Euprymna scolopes*, and is thought to provide its host with protection from predators. Genomics sequence of two strains of *A. fischeri*, ES114 and MJ11 are available. A second species from this genus, *A. salmonicida*, is the causative agent of cold water vibriosis (Hitra disease) in Atlantic salmon and rainbow trout. The genome of *A. salmonicida *strain LFI1238 has been sequenced to provide information on infectivity and pathogenicity. The eleventh genome from the *Vibrionaceae *comes from *Photobacterium profundum *strain SS9. This bacterium is not known to be pathogenic and is capable of growth at pressures of up to 70 MPa. It grows best at 10 MPa and is thus classified as a piezophile. Thirty-eight genes have been identified that are needed for growth at the high pressures and low temperatures found in the deep ocean [12].

In this paper, we investigate the distribution and features of genes that have been expanded in one specific lineage of these eleven strains, whether the expanded gene is unique to one strain or otherwise. The identity of these genes can lead us to those networks whose adaptive changes correlate with environmental challenges, interesting phenotypes, or the emergence of pathogenic effects.

## Results and discussion

### The core genome of the *Vibrionaceae*

We computed the set of orthologous proteins shared by eleven strains from the *Vibrionaceae*. The results of our inter-genomic search yielded a core genome comprised 1,882 orthologous genes (Additional file 1). Not surprisingly, this is somewhat smaller than the single species core genome of 2,741 genes established for *V. cholerae *[13]. Most (93%) of these core gene clusters contained a single representative from each strain. The number of loci represented within these orthologous clusters made up from 31 to 50% of the gene complements of these strains (Table 1). This proportion is comparable to the species-level core genome of *Escherichia coli*, *Streptococcus agalactiae *and *Haloquadratum walsybi *[14] and larger than the genus-level core genome of *Streptococcus*, which, at 600 genes, represents between 25 and 33% of the coding sequences in those genomes [15]. Given that our *Vibrionaceae *core proteome is built from representatives of three genera, the degree of conservation is remarkable. It has been observed by Vitulo et al., using k-means and hierarchical cluster analysis of phylogenetic profiles of 320 prokaryotic genomes, that the number of genes shared by organisms decreases as the number of organisms considered increases [16].

**Table 1:** Genomic sequences used in the comparative analysis of *Vibrionaceae *and the calculated number of lineage specific genes in each genome. The inter-genomic search yielded a core genome comprised of 1,882 orthologous proteins

**Strains**	**Accession ID**	**No. Genes in genome**	**No. Protein coding genes**	**% core in genome**	**No. Families with LSE**		**No. LSE genes**	**% LSEs in genome**
							
					Lineage unique	Typical LSE		
***V. cholerae* N16961**	NC_002505 (chr1) NC_002506 (chr2)	4009	3887	48.86	11	16	59	1.54
								
***V. cholerae* O395**	NC_009456 (chr 1) NC_009457 (chr 2)	3998	3878	49.05	49	25	153	3.95
								
***V. parahaemolyticus* RIMD 2210633**	NC_004603 (chr1) NC_004605 (chr2)	4708	4548	42.17	24	30	109	2.40
								
***V. vulnificus *CMCP6**	NC_004459 (chr1) NC_004460 (chr2)	4796	4796	39.82	16	24	121	2.52
								
***V. vulnificus *YJ016**	NC_005139 (chr1) NC_005140 (chr2) NC_005128 (plasmid)	4897	4758	40.08	11	26	83	1.74
								
***V. harveyi *ATCC BAA-1116**	NC_009783 (chr 1) NC_009784 (chr 2) NC_009777 (plasmid)	6238	6040	31.79	97	56	665	11.01
								
***V. splendidus* LGP32**	NC_011753 (chr 1) NC_011744 (chr 2)	4604	4431	43.35	13	56	165	3.72
								
***A. fischeri *MJ11**	NC_011184 (chr 1) NC_011186 (chr 2) NC_011185 (plasmid)	4175	4039	47.49	10	17	56	1.39
								
***A. fischeri* ES114**	NC_006840 (chr1) NC_006841 (chr2) NC_006842 (plasmid)	4038	3882	49.48	3	19	50	1.29
								
***A. salmonicida* LFI1238**	NC_011312 (chr 1) NC_011313 (chr 2) NC_011314 (plasmid) NC_011315 (plasmid) NC_011316 (plasmid)	4352	3839	49.99	12	47	248	6.48
								
***Photobacterium profundum* SS9**	NC_006370 (chr 1) NC_006371 (chr 2) NC_005871 (plasmid)	5702	5489	35.20	90	87	551	10.04

When we assigned functional categories to the core genome, the most highly represented COG functional category was metabolism (36%), followed by cellular processes and signaling (21%), and then information storage and processing (18%) (data not shown). These categories represent the proteins involved in the fundamental cellular activities common to all *Vibrionaceae*. Twenty-five percent of the core genome is either poorly characterized or not classified due to the abundance of hypothetical or unknown proteins encoded in *Vibrionaceae *genomes.

### The distribution of lineage specific expansion genes in *Vibrionaceae*

Abundant duplicate genes have been identified in the eleven *Vibrionaceae *strains. Many genes exhibit lineage specific expansion, accounting for 1–11% of the whole genomes (Table 1, also see Additional file 2). *V. harveyi *has the largest proportion of gene duplications in the strains we examined with *P. profundum *very close behind. Both these strains have genome sizes that are significantly larger (16 to 43% larger in the case of *P. profundum*, and 27 to 56% larger in the case of *V. harveyi*) than the genomes of other strains in the *Vibrionaceae*. The continuing expansion of these genomes exemplifies the conventional wisdom that gene duplication, possibly along with lateral gene transfer, are the driving forces for genome diversity as well as a buffering mechanism in response to selective pressure in bacteria [1,17]. When the distribution of expansion events is viewed in terms of the core genome, it is not surprising to see that most of the expansion events involve genes that are not in the core genome. Almost by definition, the genes in the core genome are those required to meet environmental conditions met by all eleven strains, while the genes outside the core genome are those required when strains find themselves in environmental conditions unique to their lifestyle. One of the pools of laterally transferred genes found in all the strains, and that is frequently amplified in some of them, is the large integron [18]. Researchers have noted that the similarities between the *int*I genes that anchor these integrons tend to correlate with the environment in which the organisms are found, rather than on the phylogenetic relationships among the taxa [19].

**Figure 1:**
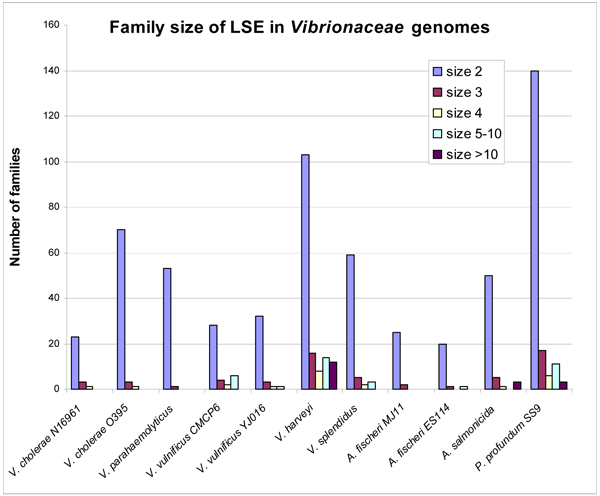
**The distribution of the size of lineage specific expanded multiple-gene families in *Vibrionaceae *strains**.

The majority of the lineage-specific expanded gene families in all the strains consist of only a few genes, which is compatible with the notion that these comparative analyses reveal only recently duplicated genes in bacterial genomes. The gene family size ranges from two to seventy-six (Figure 1). In individual *Vibrionaceae *strains, 67%–98% of the gene families are of size 2, and, collectively, gene families of 2–4 genes account for >80% of the gene families. Large gene families are rare, only found in *V. harveyi*, *A. salmonicida *and *P. profundum *(Figure 1).

By definition, each LSE family is expanded in only one of the genomes we examined. In some cases, there is no orthologous gene in any other ten genomes, and we classified these LSE families as lineage-unique. The number of such lineage-unique LSEs in each strain is shown in Table 2, along with some examples of the encoded functions. The rest of the LSE families are typical LSEs, in that they are formed from a gene for which an ortholog is found in at least one other of the genomes studied (Table 1). The lineage-unique gene families are likely to have a more significant impact on the genome they reside in because they appear to be "novel" to the pan-genome. They may have arisen from a lateral gene transfer event followed by a subsequent series of expansion events. Most of the lineage-unique LSE are hypothetical proteins with unknown functions. However, some of the lineage-unique LSEs carry out important functions, which may be related to characteristics of the host organism that distinguish it from the other members of the *Vibrionaceae *(Table 2).

**Table 2:** Examples of lineage-unique LSE families in representative *Vibrionaceae *genomes. The number in parenthesis shows the number of lineage-unique LSE families in each genome

***Strain***	**Function description**	**No. genes in families**
*V. cholerae *O395 (49)	DNA circulation protein	2
	DNA transposition protein	2
	Tail tube protein	2
	Phage virion morphogenesis protein	2
	gp27/gp16/gp05	6

*V. parahaemolyticus *RIMD 2210633 (24)	Type 1 pili subunit CsuA/B protein	2
	Dienelactone hydrolase domain protein	2
	Site-specific recombinase	2

*V. vulnificus *CMCP6 (16)	Permease of the major facilitator superfamily	2
	Transposase	2
	Cation transport ATPase	2
	Cobalamin biosynthesis protein CobW	2

*V. vulnificus *YJ016 (11)	4-hydroxyphenylacetate degradation bifunctionalisomerase	2
	CRISPR-associated protein	4

*V. splendidus *LGP32 (13)	Transposase of insertion sequence ISVisp4	18
	Protein sufB	2
	Sensory box histidine kinase/response regulator	2

*A. fischeri *MJ11 (10)	Response regulator receiver protein	2
	Acetyltransferase	3

*A. fischeri *ES114 (3)	Anaerobic glycerol-3-phosphate dehydrogenase subunit C (RebB)	9

*A. salmonicida *LFI1238 (12)	DNA-binding protein HU-alpha	2
	Phage replication repressor RstR	2
	Shufflon-specific DNA recombinase	2
	Transposase of insertion sequence families	102

*P. profundum SS9 *(90)	Selenoprotein A of glycine-reductase	2
	NosR, Regulator of nitric oxide reductase transcription	2
	Acetolactate synthase, iolD	2
	Myo-inositol catabolismprotein iolB	2
	Anaerobic dicarboxylate transporter	2
	Alpha-galactosidase (melibiase)	2
	Protease/scaffold protein	2
	Sodium: dicarboxylate symporter	3
	Methyl-accepting chemotaxis protein	3
	Reverse transcriptase/maturase family protein	3
	Helix-turn-helix XRE-family like proteins	8
	Transposase	36

### The patterns of lineage specific expansion

**Figure 2:**
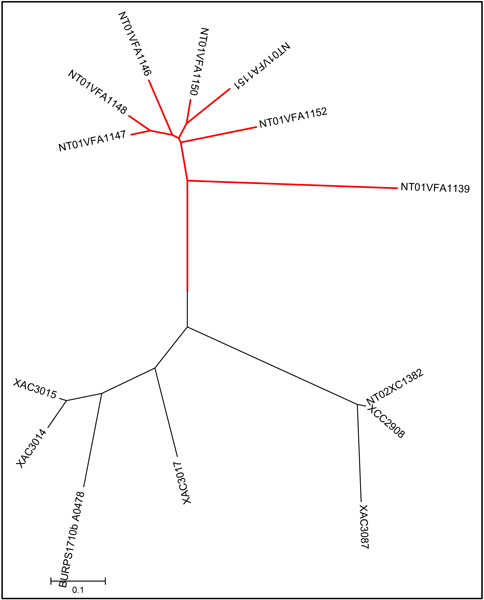
**The phylogenetic tree of anaerobic glycerol-3-phosphate dehydrogenase subunit C (rebB) genes**. The *reb*B genes in *Aliivibrio fischeri *ES114 are highlighted in red. The following genes in each species were used to infer the phylogenetic tree. *Aliivibrio fischeri *ES114: NT01VFA1139, NT01VFA1146-1148, and NT01VFA1150-1152. *Burkholderia pseudomallei *1710b: BURPS1710b_A0478. *Xanthomonas axonopodis *pv. citri 306: XAC3014, XAC3015, XAC3017, and XAC3087. *Xanthomonas campestris *8004: NT02XC1382. *Xanthomonas campestris *pv. campestris ATCC 33913: XCC2908.

Two distinct patterns of lineage specific expansion have been observed. The first type involves a single gene and can yield two or more copies in a consecutive order, a result we term a contiguous single gene expansion event. For example, two adjacent copies of a sensory box sensor histidine kinase (VC1084 and VC1085) are found in *V. cholerae *N16961. Such an expansion in the components of the two-component signal transduction networks probably aids the pathogen's response to the novel environmental conditions that it encounters. Another striking example is the occurrence of seven copies of a putative anaerobic glycerol-3-phosphate dehydrogenase subunit C (Reb) gene that is seen in *A. fischeri *ES114 (with locus IDs NT01VFA1139, NT01VFA1146-1148, and NT01VFA1150-1152), but not in other sequenced *Vibrionaceae *genomes (Figure 2). The encoded proteins are truncated (87–102 amino acid residues) versions of the full length (423 aa) C-subunit. Six genes are arranged on one strand immediately adjacent to one another, while the seventh is on the opposite strand about 15 kbp upstream. It is unlikely that these copies preserve intact enzyme functions, as they have diverged significantly from the intact enzyme. However, the six neighboring copies have maintained a sequence homology of 85 to 92%, which may be evidence of a selective pressure that is maintaining some vestigial enzymatic function that is still useful to the organism. The anaerobic respiratory activities catalyzed by anaerobic glycerol-3-phosphate dehydrogenase confer significant advantages to *A. fischeri *and its relatives *Photobacterium leiognathi *and *V. harveyi *[20].

**Figure 3:**
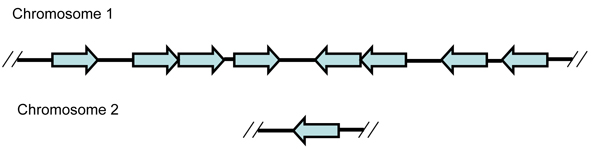
**The schematic graph of eight repeats of the four-gene block on chromosome 1 and one on chromosome 2 in *V. vulnificus *CMCP6**. Each arrow represents the four-protein block (NT01VV0474-0477), (NT01VV0913-0915, NT01VV0917), (NT01VV0920-0923), (NT01VV0964-0966, NT01VV0968), (NT01VV1054-1051), (NT01VV1158-1155), (NT01VV1365-1362), and (NT01VV1440-1437) on chromosome 1, and (NT01VVA1444-1442, 1440) on chromosome 2. NT01VV0474, NT01VV0913, NT01VV0920, NT01VV0964, NT01VV1054, NT01VV1158, NT01VV1365, NT01VV1440, and NT01VVA1444 are hypothetical cell wall-associated hydrolases, while the other proteins are conserved hypothetical proteins with unknown function.

The second pattern of lineage specific expansion we observed has a high evolutionary impact, as the expansion occurs on a larger scale, involving blocks of genes rather than a single gene. These blocks may form modules capable of related functions (Table 3). For example, in *V. cholerae *N16961 we see at least three block duplications. In one case, two pairs of paralogous genes on chromosome 2 [(VCA0393 and VCA0394) and (VCA0437 and VCA0438)] are the result of a discontiguous block duplication. The largest duplicate block in *V. cholerae *N16961, in this case a contiguous duplication, is composed of five pairs of paralogs (VC1466-VC1470 and VC1472-VC1476), which includes one pair of plasmid replication proteins, two pairs of helix-turn-helix proteins, and two pairs of hypothetical proteins. Extensive discontiguous block duplication of a four-gene block has also been identified in the *V. vulnificus *CMCP6 genome (Figure 3). Nine such blocks are located on both chromosomes, with eight blocks on chromosome 1, and one block on chromosome 2. The orientation of the eight four-gene blocks on chromosome 1 also suggests that a gene inversion occurred after the series of gene duplication events (Figure 3). Three of the duplicated genes have unknown conserved hypothetical functions, while the fourth gene is a hypothetical cell wall-associated hydrolase with no significant similarity to any known cell wall-associated hydrolase COG. A Blast search against the non-redundant protein sequence databases at NCBI found similar sequences in six other *Vibrio *species, including *V. cholerae *strains 2740–80, MAK 757, MZO-3, 623–39, and AM-19226, in *V. harveyi *strain HY01, and in *V. parahaemolyticus *strains AQ3810 and 16. There were no hits in the genera *Photobacterium *or *Aliivibrio*.

There are numerous discontiguous block expansions in the *V. cholerae *N16961 integron located on chromosome 2 (VCA0291-VCA0506). It is thought that the genes in this region provide a rich reservoir of functions that help *V. cholerae *adapt to diverse environments [19,21]. The genes falling into this region include toxin-antitoxin system genes (see below), histone acetyltransferases, metabolic enzymes, etc. [18], and many of the examples from *V. cholerae *discussed here involve genes from the integron region. These duplications are unusual in several ways. Firstly, the initial gene is acquired by a type of lateral gene transfer that involves recruitment of a cassette carrying the gene from the environment, so they are most often the seeds for lineage-unique duplications. Secondly, the duplications are often discontiguous block duplications. As a cassette does not usually include a promoter for the gene it carries, it is thought that transcription proceeds from the *int*I gene, implying that the duplicated genes will undergo a change in expression level depending on whether the genes move closer to or further from the *int*I promoter. Some of the few genes that do have their own promoters include the antitoxin/toxin genes discussed below. It is thought that, in addition to the roles discussed below, these genes may act to stabilize the integron, preserving the genes it carries from deletion [22].

**Table 3:** The Lineage Specific Expansion (LSE) block duplications in the representative *Vibrionaceae *genomes. Block size describes the number of genes in a chromosomal region that has been duplicated together

**Strains with LSE**	**Block size**	**Blocks**
***V. cholerae*** **N16961**	2	(VCA0393-0394)|(VCA0437-0438)
	5	(VC1466-1470)|(VC1472-1476)
	3	(VCA0347-0349)|(VCA0503, VCA0504, VCA0506)
***V. cholerae *O395**	3	(VC0395_A0693, VC0395_A0695-0696)|(VC0395_A0744, VC0395_A0742-0741)
	3	(VC0395_0509-0511)|(VC0395_A1063-1061)
	38	(VC0395_A0649-0692)|(VC0395_A0788-0745)
***V. parahaemolyticus* RIMD 2210633**	2	(NT01VP1132-1133)|(NT01VP1873-1874)
	2	(NT01VP1333, NT01VP1335)|(NT01VPA0972, NT01VPA0968)
	2	(NT01VP1536-1537)|(NT01VP1539-1540)
	2	(NT01VP1626-1627)|(NT01VP1769-1770)
	3	(NT01VP0604-0606)|(NT01VPA0408-0410)
	18	(NT01VP1478-1485, NT01VP1487-1497)|(NT01VPA0838-0855)
***V. vulnificus *CMCP6**	4	(NT01VV0474-0477)|(NT01VV0913-0915, NT01VV0917)|(NT01VV0920-0923)|(NT01VV0964-0966, NT01VV0968)|(NT01VV1054-1051)|(NT01VV1158-1155)|(NT01VV1365-1362)|(NT01VV1440-1437)|(NT01VVA1444-1442, NT01VVA1440)
	4	(NT01VV3112-3114, NT01VV3119)|(NT01VVA0694-0697)
	4	(NT01VV0002-0003, NT01VV0005-0006)|(NT01VVA0687, NT01VVA0689, NT01VVA0691-0692)
	2	(NT01VV2332-2333)|(NT01VV2357-2358)
***V. vulnificus* YJ016**	5	(NT02VVA0759-0763)|(NT02VVA2090-2094)
	4	(NT02VV0780-0781, NT02VV0783-0784)|(NT02VV0785-0786, NT02VV0788-0789)
	2	(NT02VVA0147-0148)|(NT02VVA2234-2235)
***V. harveyi *ATCC BAA-1116**	2	(ABU69553-69554)|(ABU73794-73795)
	2	(ABU71950-71951)|(ABU75062-75061)
	2	(ABU70467-70468)|(ABU71543-71542)
	3	(ABU71780-71782)|(ABU74683-74685)
	5	(ABU72633-72637)|(ABU73725-73721)
***V. splendidus *LGP32**	2	(CAV17464-17465)|(CAV17502-17503)|(CAV17514-17515)|(CAV19152-19153)|(CAV19489-19488)|(CAV19545-19546)|(CAV20149-20150)|(CAV20289-20288)|(CAV25337-25336)
***A. fischeri* ES114**	2	(NT01VF1641-1642)|(NT01VF1779-1780)
	7	(NT01VF0551-0553, NT01VF 0555-0558)|(NT01VFA0221-0223, NT01VFA0225-0228)
	2	(NT01VF0957-0958)|(NT01VFA1114-1115)
	7	(NT01VFA0159, NT01VFA0193-0198)|(NT01VFA0792, NT01VFA0789-0784)
***A. salmonicida* LFI1238**	2	(CAQ77717-77718)|(CAQ81906-81907)
	2	(CAQ78457-78458)|(CAQ78712-78711)
	3	(CAQ77862, CAQ77864-77865)|(CAQ77931, CAQ77929-77928)
***P. profundum* SS9**	2	(CAG23174-23175)|(CAG23550-23551)
	2	(CAG22334-22335)|(CAG22350-22351)
	2	(CAG19995-19996)|(CAG20004-20005)
	2	(CAG19407-19408)|(CAG20138-20139)
	3	(CAG19719-19721)|(CAG23185-23183)
	4	(CAG21144-21147)|(CAG21156-21160)
	7	(CAG19764-19770)|(CAG23161-23154)

### Functional categories that have undergone lineage specific expansion

The gene families that show lineage specific expansion may play important roles in defense and pathogenesis mechanisms as well as in the fundamental life cycle of *Vibrionaceae *species (Additional file 2). The existence of multiple copies of transporters, starvation response proteins, and sensor proteins seen in these strains reflects not only the evolutionary history of bacterial exposure to various stresses in the aquatic ecosystem, such as limited food supply, changes in ion concentration, and temperature swings, but also the need to interact effectively with the host. Some examples, drawn from the families our analysis uncovered, follow.

#### (i) Regulation of information processing, metabolism and cellular processes

Several gene families that function in regulation of information storage and processing, metabolism and cellular processes and signaling are seen to be expanded in specific lineages.

##### (1) Transcription

Two-component regulatory systems control many aspects of physiology, including transcription, and some of these two component transcriptional regulatory systems are expanded in *V. cholerae*, *A. fischeri*, and *V. parahaemolyticus*. They are thought to regulate diverse responses involving, for example, nitrogen acquisition and assimilation, aerobic respiration, adaptation to pH and osmolarity changes, virulence related to secretion systems, toxin production, and adherence factors. Several histone acetyltransferases (HATs) have been expanded in *V. cholerae*. Like the toxin-antitoxin system genes discussed above, these genes are found within the integron located on chromosome 2. The histone acyltransferases form an ancient family that has a role in organizing the chromosomes in Eukaryotes. In bacteria they may play a regulatory role, analogous to the integration host factor, which is thought to change chromosomal architectures in order to facilitate regulation of transcription of, for example, key virulence proteins in *V. cholerae *[23].

##### (2) Cell cycle control and cell division

Interestingly, two copies of the *lux*B gene encoding the luciferase beta chain are present in *A. fischeri *[24]. This enzyme, with its alpha subunit, catalyzes the reaction for luminescence. It is under positive regulation by the quorum sensing mechanism that coordinates the communication of bacteria and maintains the proper cell density within a limited growth space: luminescence may not be a trait that confers an advantage to the host when the density of bacteria is too low, as the light can be too weak and its generation simply a waste of energy for the bacteria.

Duplicate copies of genes homologous to toxin-antitoxin *stbDE *genes [25] have been identified in *V. cholerae *N16961 (with locus IDs VCA0489-0488 and VCA0478-0477 on chromosome 2) and *V. vulnificus *CMCP6 (with locus IDs NT01VV2333-2332 and NT01VV2358-2357 on chromosome 1). Typically, these pairs of genes have the potential to form a partnership in a toxin-antitoxin (TA) stability system. The toxic activity of one protein is normally repressed by its partner antitoxin. Bacteria have developed TA systems to promote their segregational stability. TA systems have been found located on plasmids or chromosomes in pathogenic bacteria, which suggests that these genes may function in virulence. In the case of plasmid genes, they may contribute to the segregational stability of a virulence plasmid. If a plasmid that carries the TA system is lost, the antitoxin decays more rapidly than the toxin, which is then free to act on its cellular targets. This results in the programmed cell death or stasis of the plasmid-free bacteria. In the case of chromosomal genes, it is possible that this system plays a role in the maintenance of the two chromosome genome characteristic of the *Vibrionaceae*. In natural environments Vibrios can exist as multicellular colonies or biofilms displaying coordinated cellular processes and it has been postulated that some cells in this situation will lose one of the chromosomes, becoming non-viable drone cells that can contribute to resource harvesting, but not to resource consumption [3]. The chromosomal TA systems thus tune the physiology of the bacterial cells in response to external environments, and by inducing either reversible bacteriostasis or apoptosis [26] can contribute to the overall health of the community.

##### (3) Signal transduction

Another role of two-component regulatory systems is in signal transduction. Such systems are composed of histidine sensory protein kinases (HPKs) and response regulators (RRs), and constitute key players in the mechanism by which bacteria sense and respond to changes in their environment. We have found three copies of a hypothetical sensory box sensor histidine kinase in *V. cholerae *N16961 (with locus IDs VC1084, VC1085 and VCA0719). It is likely they are involved in the signal transduction into cells; however, without identifying the response regulator partners in the two-component system, it would be difficult to determine the function of these histidine kinases.

Another example of signal transduction gene expansion is a family, seen only in *V. cholerae *N16961, of proteins that carry the GGDEF domain: proteins VC1372 and VC2224. 3', 5'-cyclic diguanylic acid (cyclic di-GMP) is an intracellular signal used in a wide variety of bacteria. Proteins carrying the GGDEF domain in bacteria play an important role in the synthesis of cyclic di-GMP, and are often linked to a regulatory domain such as the EAL domain, which participates in the degradation of the same compound. In *V. cholerae*, cyclic di-GMP regulates various processes, such as biofilm formation, virulence and transition from persistence in the aquatic environment to survival in the human gastrointestinal tract [27-30]. Proteins with a GGDEF domain and capable of modulating cyclic di-GMP concentration are quite common in the *Vibrionaceae *genomes, including the *V. cholerae *N16961 genome [31], but VC1372 and VC2224 probably represent a subset of the GGDEF-containing proteins with a role that is unique to the situations faced by *V. cholerae*.

Bacteria exhibit chemotactic responses to different substrates, such as sugars, amino acids, and dipeptides. Those transmembrane signaling responses are mediated by methyl-accepting chemotaxis proteins (MCPs). Based on signaling and adaptation domain length and sequence conservation, there are seven major MCP classes [32]. A large-scale comparative genomics analysis reveals existence of multiple copies of MCPs in *Vibrionaceae *genomes. It is noteworthy that MCPs in *A. fischeri *ES114 tend to be duplicated in tandem repeats. A couple of neighbor MCPs are located close to each other on the chromosome (with locus IDs NT01VFA0459-0460 and NT01VFA0171-0172).

##### (4) Cell wall/membrane/envelope biogenesis

The lipocalins form a family of small proteins (15–25 kDa) first described in eukaryotes and later in Gram-negative bacteria. Bacterial lipocalin Blc plays a role in storage or transport of lipids and thus is necessary for outer membrane maintenance [33]. There are four *blc *genes *blc-1*, *blc-2*, *blc-3*, and *blc-4 *in *V. cholerae*.

#### (ii) Pathogenesis, virulence, and defense

Various gene families that have been implicated in virulence, pathogenesis and defense exhibit lineage specific expansion. Different types of toxins are found in pathogenic *V. cholerae *and *V. vulnificus*, as well as in non-pathogenic *A. fischeri*. The role of toxins in pathogenic strains is obvious, while their role in the non-pathogenic symbiont *A. fischeri *may be related to squid-bacterium communication and act to ensure a beneficial outcome for the host-bacterium mutualism [5,34].

##### (1) Multidrug efflux pump

The *vce*CAB (*vce*) operon encodes the multidrug resistance efflux pump VceCAB (VCE), which contributes to resistance of *V. cholerae *to carbonyl cyanide m-chlorophenylhydrazine (CCCP), deoxycholate, and pentachlorophenol [35]. VceR, a TetR-type repressor, represses *vce*CAB operon by binding to a 28 bp inverted-repeat within the *vce*R*-vce*C intergenic region, and positively autoregulates its own expression [36,37]. The remnant of the *vce*CAB operon (*vce*AB) is identified in other *Vibrionaceae *such as *V. vulnificus *strains, in *V. parahaemolyticus *and in *A. fischeri*. Furthermore, there are two copies of *vce*AB found in non-pathogenic *A. fischeri *ES114 (with locus IDs NT01VF0957-0958 on chromosome 1 and NT01VFA1114-1115 on chromosome 2). This is consistent with our finding of extensive block duplications involving both chromosome 1 and 2 in *A. fischeri *ES114.

*V. parahaemolyticus *has a unique energy metabolism mechanism and thus requires Na^+ ^for its growth. *V. parahaemolyticus *not only possesses a primary respiratory Na^+^-pump, Na^+^-coupled membrane processes, and an Na^+^-driven flagella motor, but also has Na^+^/drug transporters [38]. We found a Na^+ ^driven multidrug efflux pump/adhesin gene duplicated in *V. parahaemolyticus *(with locus IDs NT01VP1153 and NT01VPA0916) and in *V. splendidus *(with locus ID CAV19032 and CAV26372), while a single orthologous copy remains in *V. vulnificus*, *V. cholerae, V. harveyi, A. salmonicida*, *and P. profundum *SS9. No orthologous gene was identified in *A. fischeri *ES114, which is consistent with the symbiotic environment it lives in. However, an orthologous copy exists in *A. fischeri *MJ11, implying the gene was lost in strain ES114.

##### (2) Transport systems

A number of transporter families that were previously reported to mediate drug resistance and bacteria defense, including the multidrug resistance ABC transporter family and the major facilitator family, show radiation in a single taxon. In *V. cholerae *N16961, we found two genes, VC1391 and VC1597, which share high sequence similarity, contain a conserved MFS-1 domain, and likely belong to the sugar transporter superfamily [39]. A Blast search against the non-redundant sequence database found similar sequences in other strains of *V. cholerae*, but not in other *Vibrionaceae *species, except a single gene copy in *V. angustum *S14. These *V. cholerae*-unique genes appear to be involved in multidrug transport and may relate to the pathogenesis of *V. cholerae*. In *A. fischeri *ES114, we observed a duplicated multidrug resistance protein B gene (with locus IDs NT01VF0958 and NT01VFA1115), but did not see this duplication in the other strains.

##### (3) Iron acquisition

Iron scavenging is important for host-associated bacteria, as this nutrient is almost always limiting. In *A. fischeri *ES114, a gene block on chromosome 2 with at least seven genes has been duplicated and oriented in an inverted order. An ABC-type Fe^3+^-hydroxamate transport system with 3 genes, including ATPase component, periplasmic component and permease component (with locus IDs NT01VFA0159-0161 and NT01VFA0792-0790) is duplicated and located in these blocks. These ferrichrome utilization genes (*fhu *loci) are coordinately regulated in response to iron availability [40]. We also found biopolymer transport proteins TolQ-TolQ-TolR (with locus IDs NT01VFA0194-0196 and NT01VFA0788-0786) duplicated in *A. fischeri *ES114. Similar LSE blocks were observed in strain MJ11. The *tol *genes were first described in *Escherichia coli*, and more recently in several other species. They are involved in the pathogenesis of *E. coli*, *Haemophilus ducreyi*, *V. cholerae *and *Salmonella enterica *[41]. The *tol*-*pal *genes have been shown to be required for maintaining the outer-membrane integrity of Gram-negative bacteria. The inner membrane TolA protein together with the outer membrane lipoprotein Pal forms a transmembrane link in which TolA is energized. Both TolQ and TolR proteins are essential for the TolA-Pal interaction and TolA energization [42].

##### (4) Adhesion

Pili are expressed on cell surface of Gram-negative bacteria and mediate the bacterial colonization or the attachment between host and pathogens. This attachment is a critical step in pathogenesis, thus pili are considered important virulence factors in many pathogenic bacteria [43]. Currently, four recognized types of pili have been found in Gram-negative bacteria. In the *A. fischeri *ES114 genome, ten pilus gene clusters, including eight type-IV pilus loci, have been identified [5]. The type IV-B tight adhesion pilus family is encoded within the *flp *operon, which encodes proteins responsible for the Flp fimbriae synthesis, assembly and export. Two homologous *flp *operons (with locus IDs (NT01VF0551-0553 and NT01VF 0555-0558) and (NT01VFA0221-0223 and NT01VFA0225-0228) are located on each chromosome in *A. fischeri *ES114. Two copies of this operon are also present in *A. fischeri *MJ11. A single copy of this operon is found in *V. parahaemolyticus *and *V. vulnificus*, and it is not found in *V. cholerae*. A mutation in the *flp*1 homolog on chromosome 2 of *A. fischeri *ES114 suggests that these pili are required for achieving normal colonization levels in the host light organ [44]. So it is likely that the Flp type IV pili may enhance the facultative symbiosis of *A. fischeri*, but make little or no contribution to virulence in other strains. The functional role of the *flp *operon has been studied in *Aeromonas salmonicida *subsp. *salmonicida*, a piliated bacterial pathogen of Atlantic salmon. Evidence showed that the Flp pilus made little or no contribution to virulence, while a second type IV pilus system, Tap, made a moderate contribution to virulence [45].

## Conclusion

Our analysis of gene duplication and lineage specific expansions in the *Vibrionaceae *clearly shows that this evolutionary mechanism is a major force behind genome diversification within this taxon. Two modes of expansion, single gene expansion and block expansion, are in play, and while the former is more common, the latter probably has a greater impact on the evolution of the species in this taxon. When we looked at which genes were being amplified, we first noticed that some of these genes are unique to the strain in which the LSE was observed. *V. cholerae *O395 was especially rich in these lineage-unique expansion events – over 67% of the observed expansion events were of this type. One possible explanation of this phenomenon lies with the integrons found in the chromosomes of each strain; *V. cholerae*'s integron, while not the longest, has the highest number of different gene cassettes. These environmentally acquired genes appear to be a source of many of the duplications seen in this strain. *V. harveyi *and *P. profundum *also have large portion of lineage-unique expansions. The genes amplified included genes known to be involved in the regulation of expression of not only genes involved in host colonization but also genes that help these strains survive in the environment outside the host. The amplified genes also included those encoding products directly involved in host colonization, such as fimbriae-related genes, genes involved in evasion of host defenses and genes involved in the maintenance of chromosomal DNA. It must be remembered that any discussion of gene gain has to include a mention of gene loss: without an appropriate outgroup and phylogeny, it is hard to determine whether extra genes that appear to have been gained in a particular lineage might have that appearance due to multiple gene losses in other lineages.

## Methods

### Data

We collected the complete genomes of eleven *Vibrionaceae *species (Table 1). The Genbank annotation was integrated with genome information collected from the J. Craig Venter institute's (JCVI) Comprehensive Microbial Resources Genomics database  and NCBI .

### Sequence similarity search and identification of LSE families

To identify the presence of orthologous and paralogous genes, we merged all proteins of eleven *Vibrionaceae *genomes and conducted an exhaustive all-against-all BLASTP search; genes were defined as orthologous or paralogous if (1) they had a FASTA E-score < e-10; (2) their similarity I was ≥ 30% if the length of the alignable region L ≥ 150 amino acid residues, or I = 0.01n + 4.8L(-0.32(1+exp(-L/1000))), if L <150 aa, where n = the number of sequences); (3) the length of the alignable region between the two sequences was >50% of the longer protein [46]. A Markov cluster algorithm, OrthoMCL, was used to cluster genes into gene clusters [47]. The gene clusters contain the orthologous and paralogous genes from different genomes.

Multiple alignments of each clusters were obtained by the program ClustalX [48] and T-coffee [49], followed by manual inspection and editing. Phylogenetic trees were inferred by the neighbor-joining method [50], using MEGA4 [51]. The inferred phylogenetic relationships were used to detect the orthologous and paralogous genes in each cluster. The clusters with paralogous genes (duplicated copies from the same genome) generated subsequent to the divergence of *Vibrionaceae *lineages analyzed are defined as lineage-specific expansions (LSE) in each of eleven *Vibrionaceae *lineages.

### Functional classification analysis

A hierarchical functional classification was performed for each *Vibrionaceae *sequence by searching against the Clusters of Orthologous Groups (COG) database [52]. The classification of specific supergene families including transporters, kinases, and proteases was based on the standard nomenclature defined in the Transporter Classification (TC) system [53], the Kinase Classification System [54], and the Merops Peptidase Database [55].

## List of abbreviations used

CCCP: carbonyl cyanide m-chlorophenylhydrazine; COG: Clusters of Orthologous Groups; HAT: histone acetyltransferase; HPK: histidine sensory protein kinase; JCVI: the J. Craig Venter institute; LSE: lineage-specific expansion; MCP: methyl-accepting chemotaxis protein; TA: toxin-antitoxin; TC: Transporter Classification

## Competing interests

The authors declare that they have no competing interests.

## Authors' contributions

YW, JG, and TGL conceived and designed the study. JG and YW performed bioinformatics data analysis, and drafted the manuscript. TGL participated data analysis and interpretation and edited the manuscript. JN performed pathway analysis with the help from AM and SAR. HC wrote the scripts for analysis. All authors read and approved the final manuscript.

## Supplementary Material

Additional file 1 **Core genes in eleven *Vibrionaceae *strains**. A core genome of eleven *Vibrionaceae *genomes comprised of 1,882 orthologous groups is listed. Brief descriptions of predicted gene functions and COG functional classification are also included. Click here for file

Additional file 2 Genes in *Vibrionaceae *strains that show Lineage Specific Expansions (LSEs). Click here for file
